# *δ*^13^C methane source signatures from tropical wetland and rice field emissions

**DOI:** 10.1098/rsta.2020.0449

**Published:** 2022-01-24

**Authors:** James L. France, Rebecca E. Fisher, David Lowry, Grant Allen, Marcos F. Andrade, Stéphane J.-B. Bauguitte, Keith Bower, Timothy J. Broderick, Michael C. Daly, Grant Forster, Mangaliso Gondwe, Carole Helfter, Alison M. Hoyt, Anna E. Jones, Mathias Lanoisellé, Isabel Moreno, Peter B. R. Nisbet-Jones, David Oram, Dominika Pasternak, Joseph R. Pitt, Ute Skiba, Mark Stephens, Shona E. Wilde, Euan G. Nisbet

**Affiliations:** ^1^ Department of Earth Sciences, Royal Holloway, University of London, Egham, UK; ^2^ British Antarctic Survey, Natural Environment Research Council, Cambridge, UK; ^3^ Centre for Atmospheric Science, Department of Earth and Environmental Science, University of Manchester, Manchester, UK; ^4^ Laboratory for Atmospheric Physics, Institute for Physics Research, Universidad Mayor de San Andres, Campus Universitario, Cota-Cota Calle No. 27, La Paz, Bolivia; ^5^ Department of Atmospheric and Oceanic Sciences, University of Maryland, College Park, MD, USA; ^6^ FAAM Airborne Laboratory, National Centre for Atmospheric Science, Cranfield, UK; ^7^ Consulting Geologist, 19 Jenkinson Road, Chisipite, Harare, Zimbabwe; ^8^ Department of Earth Sciences, University of Oxford, South Parks Road, Oxford, UK; ^9^ National Centre for Atmospheric Science, Centre for Ocean and Atmospheric Sciences, School of Environmental Sciences, University of East Anglia, Norwich, UK; ^10^ Okavango Research Institute, University of Botswana, Maun, Botswana; ^11^ UK Centre for Ecology and Hydrology, Atmospheric Chemistry and Effects, Bush Estate, Penicuik EH26 0QB, UK; ^12^ Department of Biogeochemical Processes, Max Planck Institute for Biogeochemistry, Jena 07745, Germany; ^13^ Climate and Ecosystem Sciences Division, Lawrence Berkeley National Laboratory, Berkeley, CA, USA; ^14^ Wolfson Atmospheric Chemistry Laboratories, Department of Chemistry, University of York, Heslington, UK; ^15^ School of Marine and Atmospheric Sciences, Stony Brook University, Stony Brook, NY, USA; ^16^ School of Chemistry, Environmental and Life Sciences, Faculty of Pure and Applied Sciences, University of The Bahamas, Nassau, Bahamas; ^17^ Department of Environmental Science, Faculty of Science, University of Botswana, Gaborone, Botswana

**Keywords:** methane, tropical wetlands, climate, greenhouse gas

## Abstract

The atmospheric methane (CH_4_) burden is rising sharply, but the causes are still not well understood. One factor of uncertainty is the importance of tropical CH_4_ emissions into the global mix. Isotopic signatures of major sources remain poorly constrained, despite their usefulness in constraining the global methane budget. Here, a collection of new *δ*^13^C_CH_4__ signatures is presented for a range of tropical wetlands and rice fields determined from air samples collected during campaigns from 2016 to 2020. Long-term monitoring of *δ*^13^C_CH_4__ in ambient air has been conducted at the Chacaltaya observatory, Bolivia and Southern Botswana. Both long-term records are dominated by biogenic CH_4_ sources, with isotopic signatures expected from wetland sources. From the longer-term Bolivian record, a seasonal isotopic shift is observed corresponding to wetland extent suggesting that there is input of relatively isotopically light CH_4_ to the atmosphere during periods of reduced wetland extent. This new data expands the geographical extent and range of measurements of tropical wetland and rice *δ*^13^C_CH_4__ sources and hints at significant seasonal variation in tropical wetland *δ*^13^C_CH_4__ signatures which may be important to capture in future global and regional models.

This article is part of a discussion meeting issue ‘Rising methane: is warming feeding warming? (part 2)’.

## 1. Introduction

The atmospheric methane (CH_4_) burden began again increasing in 2007, after some years of stability, and the growth rate accelerated in 2014 [1,2]. Concurrently, atmospheric methane's *δ*^13^C_CH_4__ has trended towards lighter (^13^C-depleted) values, implying a significant shift in the balance of sources and sinks of CH_4_ [2]. Several hypotheses have been postulated for the cause of the isotopic shift and can be summarized as one or a combination of the following: (i) a change in the oxidative capacity of the atmosphere [3], (ii) changes in the relative strengths of anthropogenic sources, such as changes to agriculture, waste and fossil fuel emissions with an overall net effect of increasing emissions (e.g. [4,5]) (iii) an increase in natural sources such as wetlands, potentially as a feedback effect from regional climatic change (e.g. [1]). There is still a large gap between top down and bottom up CH_4_ total global emissions calculations, with much of the uncertainty within the wetlands and other natural emissions categories [6].

The stable isotopic composition of methane can be a very useful diagnostic tool as *δ*^13^C_CH_4__ (and *δ*D_CH_4__) varies depending upon the processes involved in production and transportation prior to release to the atmosphere (e.g. [7]). In general terms, background air is measured as *δ*^13^C_CH_4_ _∼ −47‰, and global bulk CH_4_ inputs are estimated at approximately −53‰ [2]. The discrepancy between global input average and global background average is due to fractionation from sinks, with OH destruction of CH_4_ expected to be responsible for the majority of the 6‰ shift [8]. Biogenic sources are depleted in ^13^C, with *δ*^13^C_CH_4__ signatures in the −70 to −50‰ range for sources such as ruminants [9], wetlands [10] and rice fields [11]. Thermogenic and pyrogenic sources, such as fossil fuel emissions, biomass burning and geological seeps are generally enriched in ^13^C, with *δ*^13^C_CH_4__ signatures as enriched as −15‰ for some biomass burning events [12]. Extreme *δ*^13^C_CH_4__ variability exists on very local scales due to variation in vegetation cover, hydrology, microbial communities, etc.; however, bulk emissions from wide-area wetlands do appear to give stable *δ*^13^C_CH_4__ signatures [13] which are suitable as inputs into models.

Recent studies using global models of CH_4_ isotopes to discern global sources of CH_4_ have demonstrated the need for better spatial resolution of *δ*^13^C_CH_4__ source signature data. This is needed to reduce significant biases from assumptions of geographically invariable *δ*^13^C_CH_4__ source signatures [10,14]. Much of the knowledge gap is located in the tropical regions, with very little information for Africa and South America in particular. Previously, Sherwood *et al*. [15], Brownlow *et al*. [16] and Feinberg *et al*. [14] have collated various aspects of the tropical *δ*^13^C_CH_4__ source signatures together, but it is clear from these studies that there are still large regions where little to no studies have taken place.

Long-term global monitoring of *δ*^13^C_CH_4__ is primarily from background stations sampling the boundary layer at remote oceanic sites participating in the NOAA collaborative flask measurement programme. There is very little *δ*^13^C_CH_4__ isotopic information from inland tropical continental landmasses in South America, Africa or South Asia. This lack of regular measurements of *δ*^13^C_CH_4__ makes long-term and seasonal assessment of regional source input difficult to elucidate from the *δ*^13^C_CH_4__ record. Shorter term time-series records have been demonstrated to be useful in determining regional CH_4_ source input, such as natural gas leaks in central London [17] or the importance of industrial and fossil CH_4_ in Hungary [18].

In this work, we aim to extend the knowledge base of tropical CH_4_ source signatures for wetlands and rice fields from a range of field campaigns undertaken since 2016, creating a new working database from which global models can be populated with increased spatial resolution. We also show the value in long-term *δ*^13^C_CH_4__ sample collection at the Chacaltaya observatory, Bolivia, allowing us to analyse the data record for seasonal trends in CH_4_ input from the Amazonian region.

## 2. Methods

Air samples were collected as part of a range of field campaigns between 2016 and 2020, throughout two Natural Environmental Research Council (NERC) projects, MOYA (Methane Observations and Yearly Assessment) and ZWAMPS (Zambian sWAMPS—quantifying methane emissions in remote tropical settings). Samples were either collected as part of dedicated ground sampling, or as part of airborne measurements investigating specific regional emission features. Air samples were subsequently returned to Royal Holloway for *δ*^13^C_CH_4__ stable isotope analysis.

### (a) Ground-based sampling

**Figure 1.**
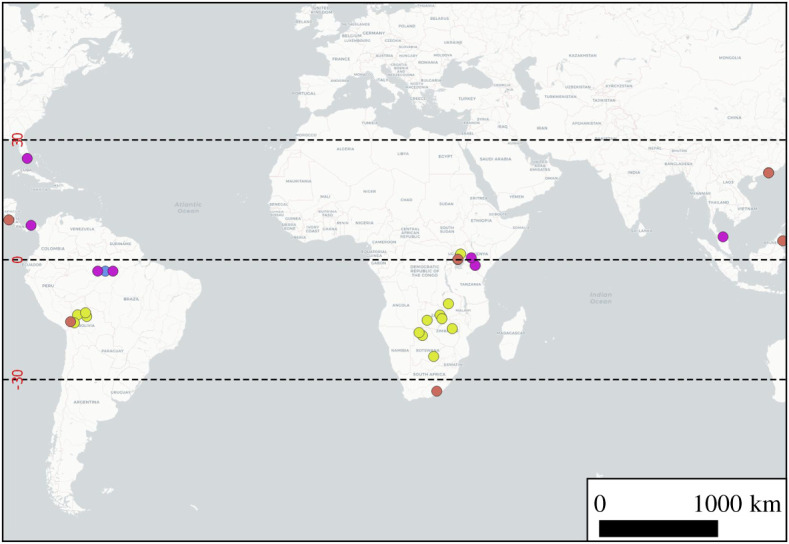
Global map to show data coverage from tropical wetland sampling campaigns with reported *δ*^13^C_CH_4__ source signatures. Yellow—this study (including aircraft campaigns), orange—Brownlow *et al*. [16], purple—summary from data collated within Sherwood *et al*. [15], blue—other. (Online version in colour.)

Ground-based sampling is targeted sampling with the intention of collecting emissions from an identified methane source. At each ground sampling location air is collected in either 3 L SKC Tedlar bags or 3 L SKC Flexfoil bags, using a battery-operated pump. Bags are filled at varying distances downwind of the targeted source being sampled, at a height of between 30 cm and 3 m height above ground. Ideally, upwind samples are also taken to provide a measure of the background airmass into which the source methane is mixing. The location of ground sample sites measured here is shown in a global context in figure 1, alongside the locations of sites used for comparison. A summary of each location of each site is shown in electronic supplementary material, table S1.

#### (i) African sampling sites

1. Botswana. There are two sites in Botswana's Okavango Delta. Ikoga, in Northern Botswana, is a village located approximately 170 km NW of Maun on the western side of the Panhandle of the Okavango Delta. Sampling took place at the water's edge of a lagoon at Ikoga Camp (an island during the wet season) (18°48′ S, 22.47′ E) and at Nxaraga (19°32′ S, 23°10′ E), located at the SW edge of Chief's Island in Moremi Game Reserve. This site is a seasonal floodplain and is typically flooded for six to eight months per year. The vegetation of the floodplain, which is dominated by C4 grasses (e.g. *Panicum repens*, *Cynodon dactylon*, *Sporobolus spicatus*), attracts many types of herbivores and is grazed for most of the year.
2. Zimbabwe. The Monovale Vlei is a seasonal wetland approximately 500 ha in extent, located in the western suburbs of the City of Harare (17°48′ S, 31°00′ E) and was declared Ramsar Site No. 2107 in 2013. Rehabilitated and protected by a local conservation society (COSMO) the open grassland fringed by miombo woodland has regained its biodiversity supporting a wide spectrum of fauna and flora, including sedges and reeds.
3. Zambia. Wetland reed beds on the Ngwerere River were sampled (15°18′ S, 28°18′ E). These are reed and papyrus-dominated wetlands north of Lusaka.
4. Uganda. Wetland marshes, fringing Lake Victoria. Samples were collected near Entebbe (0°02′ N, 32°28′ E), on the northern coast of Lake Victoria.

#### (ii) South American sampling sites

Three wetland sampling sites were chosen in northeast Bolivia's Amazonian basin, in the Llanos de Moxos region of flooded plains and savannah. The region consists of savannahs dominated by mixed C4 and C3 grasses and graminoid, aquatic and marshland plants; different types of forest islands, open forest and low spiny shrubs [19]. The region is subjected to seasonal flooding with a delay between the month with maximum precipitation, January, and the peak of maximum river discharge (February–March) [20].

1. Reyes lies in the transition between the sub-Andean Amazon forests and Llanos de Moxos. Air samples were collected in July 2016 from a wetland close to the riverbed approximately 5 km SE of Reyes town (14°20′12′′ S, −67°17′24′′ W) which represents the transition between sub-Andean Amazon forests and Moxos flooded plains.
2. Samples from the Moxos plains were taken in a 40 km transect between Trinidad city (14°48′29′′ S, 64°54′14′′ W, 130 m.a.s.l.).
3. The northern edge of Laguna Cuitarama (14°27′58′′ S, 64°50′40′′ W) during 2017 and 2019. In this area, the landscape has been modified by pre-Colombian cultures to raise beds, terraces, channels and dykes at large scale [19]. Samples were taken at the wetland edges at the end of the wet season (March 2017 and 2019) typically at the peak of flooded area in the region [20] and in May 2017.

#### (iii) Asian sampling sites

1. Hong Kong. Yi O Rice Fields are located at the southwestern tip of the largely undeveloped Lantau island (22°16′ N, 113°59′ E) 30 km from downtown Hong Kong. The rice paddies are on the floor of an uninhabited steep-sided valley and are organically cultivated using standard Chinese long-grain rice varieties. Samples were taken above fallow, freshly planted and mature crops. A series of air samples were taken from managed reedbeds at the Mai Po Wetlands (22°29′ N, 114°2′ E) at the mouth of the Sham Chun, Shan Pui and Kam Tin rivers that empty into Deep Bay along the Hong Kong S.A.R. border with Shenzhen.
2. Vietnam. Sampled rice fields are located approximately 10 km south of Ho Chi Minh City at the northeastern edge of the Mekong delta region, the primary rice cultivation area in Vietnam (10°42′ N, 106°40′ E). Samples were collected post-harvest during the dry season (late March 2019).

### (b) Airborne sampling

The airborne sampling took place over wide-area wetlands in both South America and Africa.

1. Bolivia. Airborne sampling for isotopic analysis was performed on two flight campaigns. In March 2019 air samples were obtained flying within the planetary boundary layer above the large Llanos de Moxos flooded plains [19] in Northern Bolivia. Air samples were manually collected into 3 L Tedlar bags from air taken directly into the twin-otter aircraft through an external air inlet [21]. Samples were collected at a range of methane concentrations above the wetland region, with no obvious other significant potential local sources of CH_4_ observable from the aircraft.
2. Zambia and Uganda. In February 2019, air samples were collected in stainless-steel flasks during flights above three large wetland areas in Zambia; Bangweulu, Lukanga and Kafue and in Uganda at Lake Kyoga. These campaigns were completed using the BAE-146 FAAM (Facility for Airborne Atmospheric Measurement) aircraft as the sampling platform. Sampling strategy was to collect from a range of locations and altitudes above and downwind from the wetlands, with 12–19 samples collected for each wetland.

### (c) Regional time-series sites

By contrast to the targeted wetland sampling, samples were collected for longer-term regional background measurements of methane mole fraction and *δ*^13^C_CH_4__ at sites in the Bolivian Andes and in Botswana. The sampling period from Bolivia was 6 years from 2013 to 2019 and the Botswana sampling was over 18 months from late 2016 to mid-2018. The Chacaltaya GAW Station (CHC) is located in the eastern branch of the Bolivian Andes (Cordillera Real) at 5240 m.a.s.l., 16°21′12′′ S 68°07′53′′ W. Chacaltaya is a mountain with a horizon open to the south and west facing the Altiplano (plateau of 3800 m.a.s.l.) and close to Titicaca Lake. To the east, the Amazon Basin starts at high peaks which progressively change to Yungas, sub-Andean Amazon forests and lowlands. Sampling occurred on a weekly basis from an inlet at 6 m height above ground level. The Botswana long-term site was at Modipane, a village located approximately 25 km east of the capital Gaborone in SE Botswana, which has a semi-arid environment, and is located approximately 75 km ESE of the Kalahari Desert. The sampling site at Modipane is located at the southeastern edge of the village, near to arable fields. Sampling occurred on a weekly basis using a hand-held sampling technique following the same protocol as in the ground-based sampling (§2a) with an inlet at approximately 3 m height.

### (d) Laboratory analysis

Samples collected in Tedlar bags and stainless-steel flasks are both treated to the same laboratory analysis procedure. First, the samples are analysed for methane mole fraction using a Picarro 1301 calibrated to the WMO X2004A CH_4_ scale. Samples are flushed through the Picarro for 120 s to ensure no contamination from previous sampling, and then analysed for 120 s. The mean of the 120 s of analysis (approx. 12 measurements) is calculated as the measured value. Precision is approximately 0.5 ppb for both Tedlar bags and stainless-steel flasks. Samples are then prepared for isotopic analysis (*δ*^13^C_CH_4__) by continuous-flow isotope-ratio mass spectrometry using an Isoprime Trace Gas system [22]. Samples are run in triplicate, with precision of approximately 0.05‰ (precision is determined for each sample). Values of *δ*^13^C_CH_4__ are reported on the standard international isotope VPDB (Vienna Pee Dee Belemnite) scale. An internal secondary standard is analysed through the day to allow correction for instrumental drift.

### (e) Determination of *δ*^13^C_CH_4__ source signatures

In order to determine the ^13^C_CH_4__ isotopic source signature for the various source types a Keeling plot regression is used [23]. For each location, *δ*^13^C_CH_4__ are plotted against the inverse of the CH_4_ mixing ratio to allow determination of the *δ*^13^C_CH_4__ value at the *y*-intercept which represents the *δ*^13^C_CH_4__ of the methane added to the natural background. This is represented mathematically in equation (2.1) and has been successfully applied in numerous studies to determine methane source signatures (e.g. [13,16,24]).

2.1 $$\delta^{13}{\text{C}}_{\text{a}}=c_{b}(\delta^{13}{\text{C}}_{\text{b}}-\delta^{13}{\text{C}}_{\text{s}})\times \left(\frac{1}{c_{a}}\right)+\delta^{13}{\text{C}}_{\text{s}},$$

where *c_a_* is the measured atmospheric concentration of methane, *c_b_* is the atmospheric background concentration of methane, *δ*^13^C_a_ is the measured atmospheric isotopic composition, *δ*^13^C_b_ is the background atmospheric isotopic composition and *δ*^13^C_s_ is the source isotopic composition.

For sites where multiple days or campaigns were conducted to sample the same source (such as the Hong Kong rice fields and wetlands, and the Nxaraga site in the Okavango Delta) a Miller–Tans approach was used [25] (2.2). The longer-term records of *δ*^13^C from background sites at Chacaltaya, Bolivia and from Modipane, Botswana were analysed using a Miller–Tans methodology [25] adapted for interrogating long-term periodic sampling. In a traditional Miller–Tans source determination method, a suitable background is determined for each period of sampling. As that is not feasible for periodic sampling, each point is ascribed a background sample to allow the calculation in equation (2.2). The background sample chosen is the most recent local minimum from within the last 60 days. This allows for seasonal and long-term changes to be taken into account in the *δ*^13^C and CH_4_ mixing ratio record rather than assuming a fixed atmospheric background which would be the case in using a Keeling methodology for this purpose. Miller–Tans methodology allows investigation into both the bulk isotopic input of CH_4_ to a fixed site, as well as being able to interrogate the data for seasonal and longitudinal trends.

2.2 $$\delta^{13}{\text{C}}_{\text{obs}}\times c_{\text{obs}}=\delta^{13}{\text{C}}_{\text{s}}\times c_{\text{obs}}-c_{bg}(\delta^{13}{\text{C}}_{\text{bg}}-\delta^{13}{\text{C}}_{\text{s}}),$$

Where *δ*^13^C is the measured isotopic composition of the methane, *c* is the mixing ratio of the methane, bg, obs and s refer to background, observations and source, respectively.

## 3. Results and discussion

### (a) Ground-based wetland data

The calculated source signatures are grouped into two categories—individual source signatures (wetlands, table 1; rice fields, table 2) and bulk area isotopic signatures from aircraft and regional fixed site measurements (table 3). These are presented alongside a review of previously measured tropical source signatures of the same type. The new individual studies reported here add to the limited range of previous studies, increasing the confidence of the isotopic values which should be considered for regional and global modelling inputs.

**Table 1.** Summary of tropical isotopic source signatures from ground-based sampling of wetlands along with tropical isotopic source signatures for wetlands from Brownlow *et al*. [16] and relevant data from the compilation of source signature data within Sherwood *et al*. [15]. Codes for errors quoted are s.d., standard deviation. s.e., standard error. ½ range, half of measurement range from multiple sources.

country	category	*δ*^13^C_CH_4__	error	type	reference	sampling period
Brazil	floodplain	−58.5	1.9	1 s.e.	[26]	Apr, Aug, Dec 1985–1988
Brazil	floodplain	−54	7.3	1 s.d.	[27]	July–Aug 1985–1987
Brazil	floodplain	−63.9	0.6	1/2 of range	[28]	June 1981
Kenya	lake	−48	2.5	1 s.d.	[29]	Apr 1986
Kenya	river	−54.2	0.4	1/2 of range	[29]	Apr 1986
Kenya	swamp	−61.7	0.5	1/2 of range	[29]	Apr 1986
Kenya	papyrus marsh	−31.2	n.a.	n.a.	[29]	Apr 1986
Panama	multiple sources	−61.9	3.2	1 s.d.	[30]	year-round
Thailand	river	−68.3	3.1	1 s.d.	[31]	year-round 1990–1992
Thailand	swamp	−65.4	5.6	1 s.d.	[31]	year-round 1990–1992
USA	estuary	−65.7	3	1 s.d.	[32]	Aug–Jan 1984–1985
USA	lake	−61.5	6.1	1 s.d.	[32]	Aug–Jan 1984–1985
USA	marsh	−61.7	3.6	1 s.d.	[33]	year-round 1986–1987
USA	marsh	−63.1	0.2	1 s.d.	[34]	Dec 1985
USA	marsh	−68.1	2	1/2 of range	[34]	Dec 1985
USA	marsh	−70.1	1.8	1 s.d.	[34]	Dec 1985
USA	marsh	−63.5	1	1 s.d.	[34]	Dec 1985
USA	everglade flooded marsh (oxidation)	−57.3	3.6	1 s.d.	[35]	Oct, Jan, Mar 1989–1992
USA	everglade flooded marsh (no oxidation)	−63.1	2.6	1 s.d.	[35]	Oct, Jan, Mar 1989–1992
Hong Kong	marsh	−52.3	0.7	1 s.d.	[16]	June 2016
Uganda	papyrus swamp	−53.0	0.4	1 s.d.	[16]	May 2014
Costa Rica	coastal floodplain freshwater marsh	−53.3	1.7	1 s.d.	[16]	Feb 2016
Uganda	freshwater wetland	−58.7	4.1	1 s.d.	[16]	May 2014
Bolivia	freshwater wetland	−59.7	1.0	1 s.d.	[16]	Feb 2014
Hong Kong	marsh	−60.2	0.4	1 s.d.	[16]	June 2016
Borneo	forest wetland	−61.5	2.9	1 s.d.	[16]	Aug 2015
South Africa	freshwater wetland	−61.5	0.1	1 s.d.	[16]	Dec 2014
Bolivia	seasonal wetland	−57.4	1.0	1 s.d.	this work	July 2016
Bolivia	seasonal wetland	−55.8	0.6	1 s.d.	this work	Mar 2017
Bolivia	seasonal wetland	−54.3	0.8	1 s.d.	this work	May 2017
Bolivia	seasonal wetland	−55.5	4.5	2 s.d.	this work	Mar 2019
Hong Kong	reeded wetlands	−62.7	2.1	1 s.d.	this work	Mar 2018
Uganda	lake edge wetland	−54.2	0.9	1 s.d.	this work	Jan 2019
Zambia	riverine reeded wetland	−59.6	2.0	1 s.d.	this work	Jan 2019
Zimbabwe	wetland plains	−58.3	1.7	1 s.d.	this work	Feb 2017
Zimbabwe	wetland plains	−56.2	1.9	1 s.d.	this work	Apr 2020
Botswana	seasonal wetland	−56.3	1.1	2 s.d.	this work	Aug 2017
Botswana	seasonal wetland	−31.4	5.1	2 s.d.	this work	Feb 2017

**Table 2.** Summary of tropical isotopic source signatures for rice paddies from this work and a review of previous published data. See table 1 for error abbreviations.

location	*δ*^13^C_CH_4__	error	type	reference	sampling year and information
China	−63.8	4.9	1 s.d.	[36]	1995 during growing season
Japan	−65.8	3.8	2 s.d.	[37]	1990/1991 during growing season
Japan	−63.1	4.9	1 s.d.	[37]	1990/1991 during growing season
Japan	−55.9	4.2	1 s.d.	[38]	1989 throughout season
Japan	−59.6	3.4	1 s.d.	[38]	1989 throughout season
Kenya	−59.4	1.9	1 s.d.	[29]	1986 growing season
Thailand	−54	5.9	1 s.d.	[31]	1990–1992 throughout
USA	−64.5	1	1/2 range	[39]	1991 July growing season
USA	−63.2	2.9	1 s.d.	[40]	1987 May–June growing season
Hong Kong	−58.7	0.4	1 s.d.	[16]	June 2016 growing season
Hong Kong	−59.0	0.4	1 s.d.	[16]	June 2016 growing season
Hong Kong	−59.1	0.8	1 s.d.	this work	Yi O Rice 2017 growing season
Hong Kong	−57.2	0.4	1 s.d.	this work	Yi O Rice 2018 growing season
Hong Kong	−58.2	1.7	1 s.d.	this work	Yi O Rice 2019 growing season
Hong Kong	−57.0	0.3	2 s.d.	this work	Yi O Rice 16–20 combined dataset
Vietnam	−62.4	3.0	1 s.d.	this work	Ho Chi Min City post-harvest

**Table 3.** Summary of bulk tropical isotopic source signatures using aircraft and long-term observations studies for wetlands from this work and a review of previous published data. Errors are quoted to 1 s.d. Bolivia aerial wetland studies conducted in March 2019. Zambia and Uganda aerial studies conducted in February 2019.

country	category	*δ*^13^C_CH_4__	error	type	method summary
Bolivia	bulk wetland	−58.7	1.9	1 s.d.	aircraft sampling—Keeling
Bolivia	long-term regional	−59.0	1.3	1 s.d.	Miller–Tans
Uganda	bulk wetland aerial	−52.2	1.0	1 s.d.	Keeling
Zambia	wetland bulk aerial	−59.7	0.7	1 s.d.	aircraft sampling—Keeling
Zambia	wetland bulk aerial	−60.0	1.2	1 s.d.	aircraft sampling—Keeling
Zambia	wetland bulk aerial	−62.1	2.3	1 s.d.	aircraft sampling—Keeling
Botswana	long-term regional	−55.4	4.6	1 s.d.	Miller–Tans

**Figure 2.**
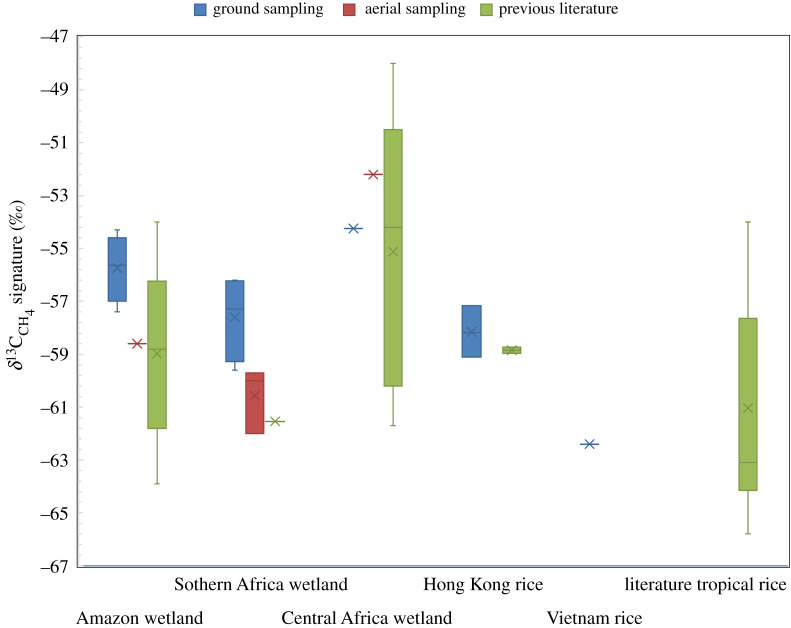
Summary box and whisker plot to allow comparison of source signatures for tropical wetlands and rice fields in this work with literature data compiled in figure 1 from within Sherwood *et al*. [15], Brownlow *et al*. [16] and Beck *et al*. [42] for rice fields and tropical wetlands *δ*^13^C_CH_4__ source signatures. (Online version in colour.)

We obtained *δ*^13^C_CH_4__ source signature values in the range of −57.4 ± 1‰ to −54.2 ± 1‰ for seasonal Bolivian wetlands, −62.7 ± 2.4‰ for reed bed Hong Kong wetlands, −54.2 ± 0.9‰ for Lake Victoria edge papyrus wetlands and −58.3 ± 1.7‰ to −56.2 ± 1.9‰ for seasonal Zimbabwe wetlands. Table 1 places the range of individual wetland *δ*^13^C_CH_4__ source signatures in the context of previously reported values. A single exceptional *δ*^13^C_CH_4__ measurement of −31.4‰ was obtained at Ikoga Camp, Botswana. The presence of such an isotopically heavy signature in a wetland sample has only been recorded once before at a Kenyan wetland dominated by papyrus [29]. This relatively ^13^C rich methane may be indicative of smoke input from a fire. Previous studies in the Okavango have reported subsurface peat fires which may explain these anomalous results [41]. Repeat sampling was undertaken at Okavango Delta sites across several seasons (electronic supplementary material, figure S1), but the data showed inconclusive source signatures from these further studies, with field observations noting the presence of burnt papyrus indicating that biomass burning events may indeed be responsible for the heavier isotope source signatures. The measured tropical wetlands here are compared with the most established database of *δ*^13^C_CH_4__ source signatures from Sherwood *et al*. [15] and Brownlow *et al*. [16] in figure 2.

### (b) Bulk wetland—aerial data collection

**Figure 3.**
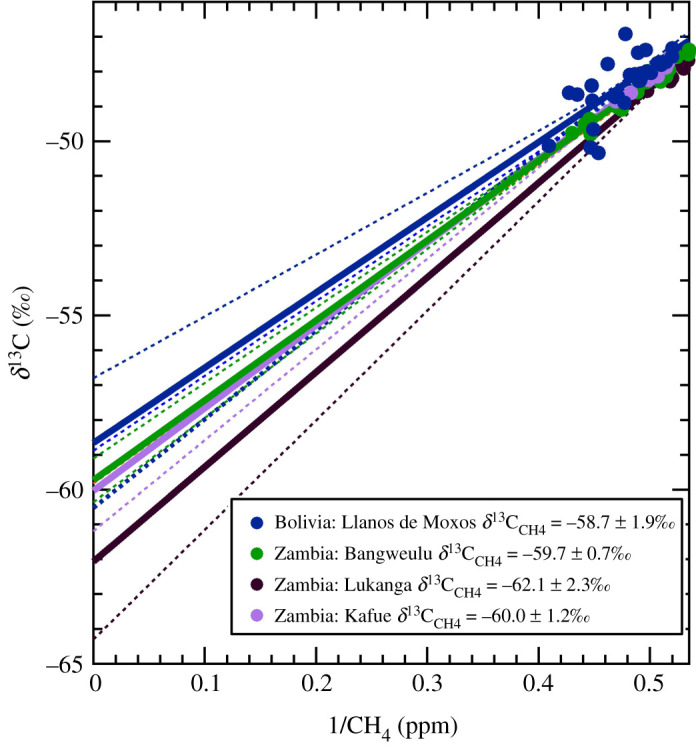
Keeling plots from aerial measurements to determine source signatures of bulk fluxes from Bolivian and Zambian wetlands. Dashed lines represent 1 s.d. confidence interval in the linear regression. Colours of the regression and uncertainties match the symbol colours of the sample locations. (Online version in colour.)

The bulk wetland studies using aerial methods appear to have a slightly more depleted source signature than those collected at wetland edges for the same wetlands in Zambia (table 3). The wetlands studied by aircraft sampling are larger and have a permanent wetland extent. The large wetlands in Zambia give a range of −62.1 ± 2.3‰ to −59.7 ± 0.7‰, the Bolivian Llanos de Moxos bulk signature is −58.7 ± 1.9‰ and the Ugandan Lake Kyoga wetland a signature of −52.2 ± 2‰. The heavier isotopic source signature reported for Ugandan wetlands may be due to differences in the ratio of C3–C4 vegetation compared to the Bolivian and Zambian wetlands, as an increase in C4 plants such as papyrus would be expected to lead to a heavier isotopic source signature compared to a C3 reeds and grasses dominated wetland [12,43]. It should also be noted that the *δ*^13^C scatter within the Keeling plots for the Llanos de Moxos and Lukanga (Zambia) may indicate that there are variable *δ*^13^C_CH_4__ inputs to the atmosphere from these large area wetlands (figure 3). The variable inputs could be due to changes in vegetation type or due to water level variation throughout the wetland, as reported for Amazonian basin wetlands in Pangala *et al*. [44]. No significant sources of CH_4_ other than wetlands were noted or seen in any of the ancillary atmospheric chemistry measurements where samples were taken for isotopic analysis during the flights.

### (c) Rice paddies

The rice paddies of Hong Kong were measured over four separate seasons from 2016 [16] to 2019. The resulting individual source signatures give a range of −59.1 ± 0.8‰ to −57.2 ± 0.4‰ and the composite Miller–Tans result gives an overall *δ*^13^C_CH_4__ source signature of −57.1 ± 0.3‰ (table 2). The lack of interannual variability in the rice signature suggests that the bulk CH_4_ atmospheric input from rice fields is consistent and that a *δ*^13^C_CH_4__ source signature of −57.1 ± 0.3‰ is consistent through time. A single snapshot study from drying Vietnam rice fields gives a source signature of −62.4 ± 3‰, which is more depleted but with considerably greater uncertainty. Previously recorded data on rice fields *δ*^13^C_CH_4__ from Brownlow *et al*. [16] and Sherwood *et al*. [15] (table 2) gives an average signature of approximately −61 ± 4‰ for all rice fields, which is consistent with the new data presented here in figure 2.

### (d) Time-series regional data

**Figure 4.**
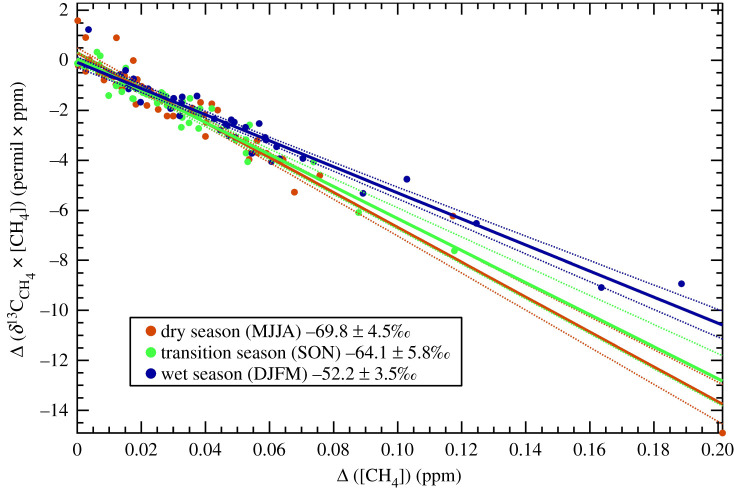
Miller–Tans interpretation of the Chacaltaya Observatory 6 year-long isotope record. Samples have been split into the generalized wet, dry and transition seasons to identify any significant variability in bulk input. Confidence intervals shown as dotted lines are 95% bands and uncertainties quoted on the source signature are to 95% confidence. (Online version in colour.)

Longer-term time-series monitoring of *δ*^13^C_CH_4__ at both Modipane, Botswana and at Chacaltaya, Bolivia are shown in electronic supplementary material, figure S2. The results of Miller–Tans analysis for these two sites shows regional bulk input of methane is dominated by biogenic sources. Bulk averaged input using all available data for Modipane is −55.4 ± 4.6‰ and −59.0 ± 1.3‰ for Chacaltaya. These results match well with their respective in-country seasonal wetland measurements of −56.3 ± 1.1‰ for Botswana (table 1) and the measurements from the extensive Bolivian wetlands during the wet season of −58.7 ± 1.9‰ (table 3). However, when the data is split into seasonal bins of wet season, dry season and transition season, the CH_4_ isotopic input measured at Chacaltaya appears seasonally dependent with much lighter isotopic input occurring in the dry and transition seasons compared to the wet season with a seasonal variability of 18‰ (figure 4). This variability indicates that there are subtleties to the seasonal regional bulk input of methane which are not currently accounted for or well understood. The dataset for Modipane only covers 18 months and is not a substantial enough dataset to infer seasonality.

**Figure 5.**
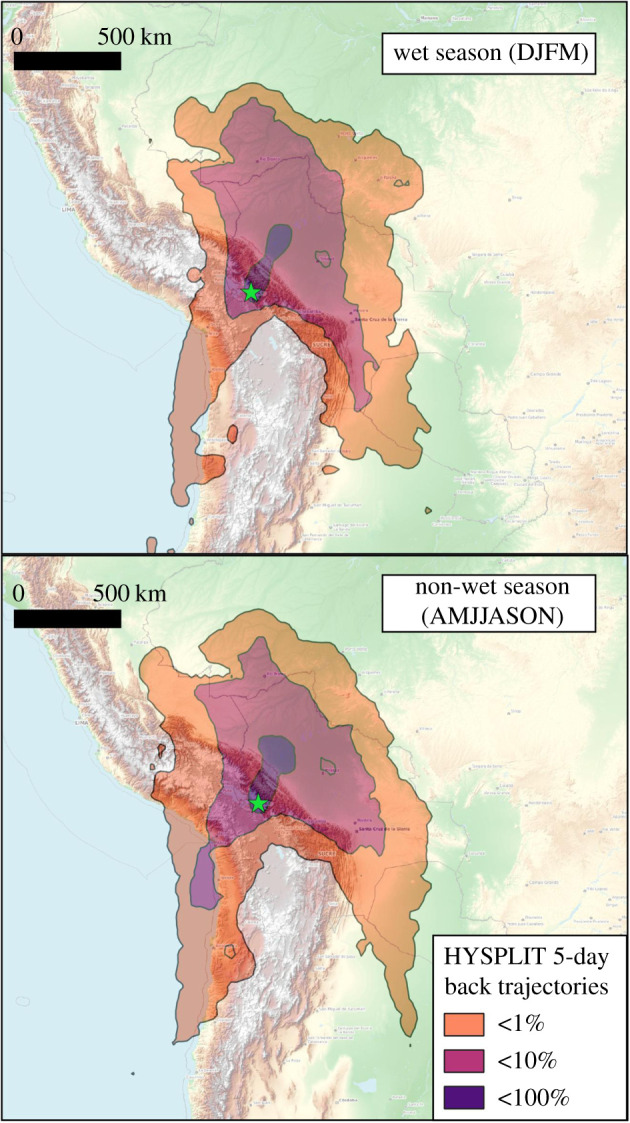
HYSPLIT back trajectory frequency analysis from the Chacaltaya observatory, Bolivia. Station altitude 5240 m.a.s.l. Trajectories created using 2.5° NCEP 6-hourly re-analysis data and are a composite of 120 h back trajectories taken every 6 h from 2014 to 2017 inclusive. Only periods where the airmass was within the lowest 1000 m of the atmosphere are considered. (Online version in colour.)

Detailed Weather Research and Forecasting (WRF) model back trajectory analysis from Chacaltaya observatory was conducted previously over a 5-year period [45]. The back trajectories show a seasonal variability in air mass origins, with a greater dry season input of air masses from the Peruvian sector, mainly from the Altiplano, and a larger input from the Bolivian lowland plains in the wet season. These features are also captured to some extent in the coarser resolution composite 4-year HYSPLIT back trajectory analysis shown in figure 5.

### (e) Potential mechanisms of seasonal variation

Seasonal shifts in the *δ*^13^C of methane likely reflect differences in wetland methane production, oxidation and emissions pathways throughout the year. The Bolivian Llanos de Moxos wetlands experience an extremely strong seasonal cycle in inundation, with inundated area changing by over an order of magnitude seasonally, as well as large interannual variability in inundation [20,46]. The resulting changes in water table depth and duration of flooding, and variation in C inputs all have the potential to influence methane production and oxidation. In diffusion dominated environments, variation in the rate of methane oxidation is likely to be a primary control on the *δ*^13^C of emitted CH_4_ [47]. However, seasonal shifts in *δ*^13^C_CH_4__ are not limited to diffusive emissions; for example, Smith *et al*. [48] also observed a seasonal shift of over 10‰ in the *δ*^13^C of CH_4_ emitted from macrophytes in the Orinoco River floodplain, correlated with strong seasonality in water level and methane emissions.

Variability in air mass origins does not explain the isotopically very negative *δ*^13^C_CH_4__ source signatures seen in the Bolivian data. Such very light values suggest some emissions come directly from biological sources without intervening methanotrophic oxidation during egress from water or soil, because methanotrophic removal selectively removes isotopically light carbon, causing selective enrichment in the remaining methane and resulting emissions. One possibility is that tree-mediated emissions in Amazonia, with a *δ*^13^C_CH_4__ source signature as light as −76.3 ± 0.9‰ [44], are a potential source of the sampled isotopically light methane. Thus, variation in the water table, and therefore the amount of oxidation of CH_4_ prior to emission may be an important control on the isotopic flux to the atmosphere from these expansive tropical wetlands.

The observed seasonal shifts in *δ*^13^C might also reflect changes in the distribution of wetland types responsible for the methane emissions. For example, the permanently inundated wetlands may differ from the seasonally inundated wetlands in key environmental parameters including pH, vegetation type and nutrient status. These environmental parameters in turn control the relative proportion of acetoclastic versus hydrogenotrophic methanogenesis, and the resulting *δ*^13^C of CH_4_ produced [49]. More ^13^C-enriched CH_4_ is expected where acetoclastic methanogenesis dominates, with more depleted CH_4_ found in nutrient poor environments where CO_2_ reduction dominates. For example, methane as depleted as −94‰ was observed in the porewater of an ombrotrophic peatland in Panama [49], and similar values have been observed in porewater in Borneo and Peru [50]. Thus, seasonal changes in *δ*^13^C could result from a changing relative contribution of different wetland types within a wetland complex throughout the year.

Additionally, there may be seasonal shifts in the primary methane emissions pathways, with varying contributions from tree stem emissions, below-ground transport of dissolved gases, surface diffusion and ebullition. Diffusive emissions are much more likely to be highly oxidized, resulting in a more enriched *δ*^13^C [47]. By contrast, ebullition, belowground transport and tree emissions offer more direct emissions pathways which may bypass opportunities for oxidation, resulting in a more depleted *δ*^13^C signature [44]. Thus, given the strong seasonality in the processes driving methane emissions from tropical wetlands, more seasonal measurements of the *δ*^13^C of methane emitted from tropical wetland complexes is needed to determine the extent to which the bulk signature is affected. Although it is not possible from the data obtained here to determine fluxes, the flux-weighted *δ*^13^C signature is not anticipated to be overlysignificantly weighted towards the wet season, with data from Miller *et al*. [51] indicating that strong enhancements in CH_4_ have been measured in the Amazon Basin in both the wet and dry seasons. Recent modelling by Tunnicliffe *et al*. [52] estimates wetland emissions for Brazil from 2010 to 2018 to be approximately twice as strong in the wet season as for the dry season. Using the Tunnicliffe *et al*. [52] monthly wetland flux estimates for weighting, a flux-weighted *δ*^13^C source signature for Chacaltya due to wetland methane is calculated as −61.5 ± 4.1‰ (equation 3.1). This compares well with the overall non-seasonally split *δ*^13^C source signature for Chacaltya determined from the long-term record of −59.0 ± 1.3‰.

3.1 $$\begin{array}{rl} \delta^{13}{\text{C}}_{\left(\text{flux–weighted}\right)} & \;=[(\delta^{13}{\text{C}}_{\text{dry}}\times \text{C}{\text{H}}_{4(\text{dry–flux})})+(\delta^{13}{\text{C}}_{\text{transition}}\times \text{C}{\text{H}}_{4(\text{transition–flux})})\\ & \;\;+(\delta^{13}{\text{C}}_{\text{wet}}\times \text{C}{\text{H}}_{4(\text{wet–flux})})]/\text{C}{\text{H}}_{4(\text{flux–annual})}, \end{array}$$

where *δ*^13^C_dry_, *δ*^13^C_transition_ and *δ*^13^C_wet_ are taken from values in figure 4 and CH_4 (dry-flux)_, CH_4 (transition-flux)_ and CH_4 (wet-flux)_) are the corresponding monthly average wetland fluxes derived for Amazon wetlands from 2010 to 2018 [52].

The importance of understanding the seasonal variation of the isotopic signal emitted to the atmosphere will be key in coupling the subsurface processes driving the production and transportation of methane within the wetlands and the methane fluxes to the atmosphere from wetlands—a key component of the global methane budget.

## 4. Conclusion

The importance of tropical wetlands to the global methane budget is well established, but the role that wetlands play in the current increase in global methane mixing ratios is still under debate. Continued work to understand the isotopic composition of wetland emissions is important to improve the accuracy of global models using *δ*^13^C_CH_4__ to determine the causes behind the recent global methane atmospheric growth. The results here show that rice emission *δ*^13^C_CH_4__ signatures appear to be very stable on an interannual basis and that generalized value of −61 ± 4‰ for rice is a reasonable model input. There appears to be good agreement in bulk wetland emissions from similar latitudes from both South America and Africa during the periods of peak wetland extent, with values of approximately −60 ± 5‰ as a recommended generalized value for the outer tropical wetlands from the wide-area aerial wetland sampling. The more papyrus rich tropical wetlands of the Okavango and Uganda appear to have an isotopically heavier *δ*^13^C_CH_4__ signature, with a bulk wetland signature of approximately −52 ± 2‰ measured above Ugandan wetlands indicating that relatively small (on a global scale) latitudinal and climatic changes in the tropics have a significant effect on *δ*^13^C_CH_4__ signatures. The values reported here for the bulk outer tropical wetland *δ*^13^C_CH_4__ signatures appear lighter than expected when comparing to the global map shown in Ganesan *et al*. [10] and also generally lighter than sampling occurring at wetland edges. As regional and global models look to use isotopic trends to better understand the global methane budget, gaps and nuance in the *δ*^13^C_CH_4__ signature records used as model inputs should not be overlooked. Continuing to fill in the unknowns in the tropical methane *δ*^13^C_CH_4__ source signature record remains a priority, as data is currently extrapolated over vast geographical ranges, especially in Central and Northern Africa and Australasia where data are incredibly limited.

The influence of seasonality in the tropical wetland *δ*^13^C signature requires further investigation, ideally through targeted, longer-term campaigns to measure time series of emissions from tropical wetlands through several seasonal cycles. The long-term data from Chacaltaya shows strong seasonal isotopic differences, but the variability in the data cannot conclusively be tied to wetland emissions. Seasonal changes to soils and sediments would be expected to result in seasonal changes to the *δ*^13^C_CH_4__ source signatures of bulk atmospheric emissions from tropical wetlands, but there is no conclusive evidence to demonstrate the effect occurs or by how much. Improving the understanding of the latitudinal variability and seasonality of wetland emissions is key to being able to isotopically balance the *δ*^13^C_CH_4__ budget in global models. Further work investigating isotopic signatures from extensive permanent and seasonal wetlands over multiple years is now an important target to allow us to understand the large-scale seasonality of wetland emissions and the processes that control them.

## Data accessibility

Data will be available via the MOYA project CEDA database (https://catalogue.ceda.ac.uk/uuid/dd2b03d085c5494a8cbfc6b4b99ca702) and via the corresponding author.

The data are provided in the electronic supplementary material [53].
